# Corrigendum: Polaronic metal state at the LaAlO_3_/SrTiO_3_ interface

**DOI:** 10.1038/ncomms10920

**Published:** 2016-02-26

**Authors:** C. Cancellieri, A. S. Mishchenko, U. Aschauer, A. Filippetti, C. Faber, O. S. Barišić, V. A. Rogalev, T. Schmitt, N. Nagaosa, V. N. Strocov

*Nature Communications* 7 Article number: 1038610.1038/ncomms10386 (2016); Published 01272016; Updated 02262016

In the original PDF version of this Article, which was published on 27 January 2016, the publication date was incorrectly given as 27 January 2015. This has now been corrected in the PDF; the HTML version of the paper was correct from the time of publication.

