# Supplementary material for: Polaronic metal state at the LaAlO3/SrTiO3 interface
Source: Nat Commun. 2016 Jan 27;7:10386. doi: 10.1038/ncomms10386 (PMC4737810; doi:10.1038/ncomms10386)
Supplement: Supplementary Information — Supplementary Figures 1-2, Supplementary Notes 1-2 and Supplementary References. [file ncomms10386-s1.pdf]

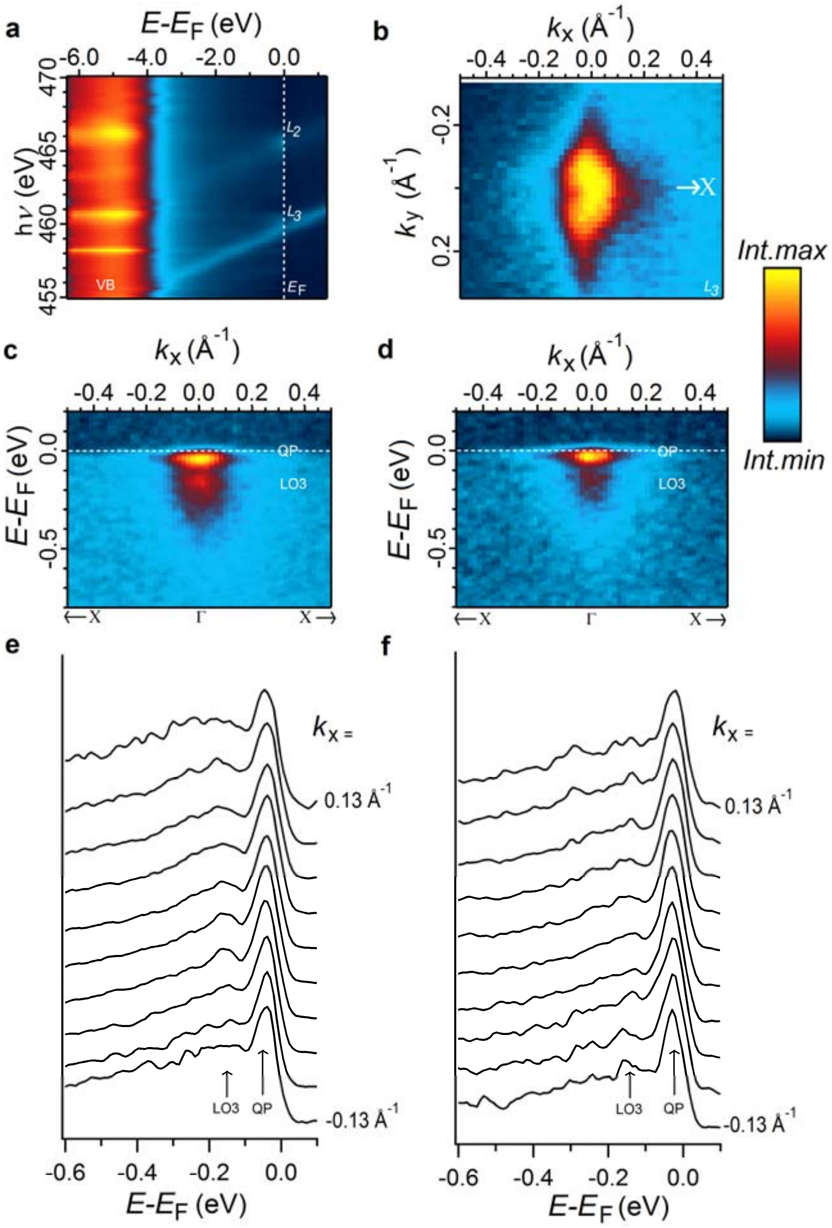

Supplementary Figure 1. Experimental low-temperature (12K) SX-ARPES results collected with *p*-polarization. a, Resonance photoemission intensity map, identifying the 2DES signal at the L_3 and L_2 resonances. **b**, FS map at the L_3-resonance, showing the d_{xz}-derived sheet. **c,d**, High-resolution ARPES images along the ΓX line at the L_3- and L_2-edges, showing the d_{xz}-derived band. The color bar indicates the intensity (Int.) maximum and minimum. **e,f** A series of (normalized) EDCs extracted from **c,d**, respectively, at the indicated k_x-values. The two curves at the bottom show EDCs integrated over the whole BZ in the k_x-direction. The *p*-polarization data confirms the characteristic PDH spectral structure manifesting a polaronic metal state formed by the hard LO3 phonon.

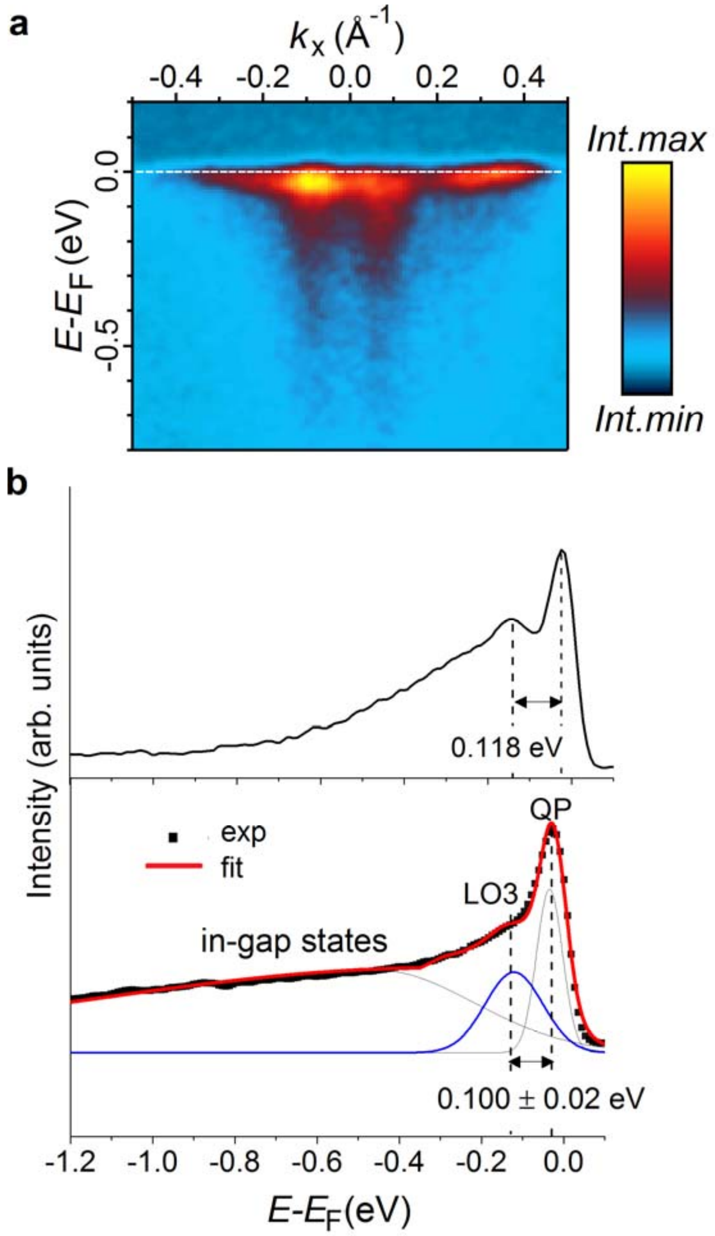

Supplementary Figure 2. Experimental SX-ARPES results on OxD-LAO/STO samples. a, High-resolution ARPES image along the ΓX line at the L_3-edge, showing larger k_F and thus n_s. The color bar indicates the intensity maximum and minimum. **b** The corresponding k_x-integrated EDC (*bottom*) compared with that of the standard sample (*top*). Constant energy of the hump proves its polaronic origin, and its scaling down illustrates a possibility to manipulate the EPI through the V_Os.

Supplementary Note 1

SX-ARPES data for p-polarized X-rays

Here, we present our SX-ARPES data acquired at 12K with *p*-polarized X-rays, parallel to the *s*-polarization data reported in the main text, Figs. 1 and 2. We select now the d_{xz}-derived states, symmetric relative to the ΓX line of the two-dimensional BZ. The resonance map of (angle-integrated) photoemission intensity, Supplementary Figure 1a, again identifies the 2DES signal at E_F blowing up near the two Ti^{3+} L_3- and L_2-resonances, although its intensity is smaller compared to the *s*-polarization because of the missing strong d_{xy}-intensity. The FS map in Supplementary Figure 1b acquired at the stronger L_3-resonance clearly displays the elliptical d_{xz}-sheet extending in the k_y –direction and derived from the d_{xz}-derived state symmetric relative to the ΓX line. Consistently with this map, the ARPES images measured along the ΓX line at the L_3- and L_2-resonances, Supplementary Figure 1c and d respectively, display the d_{xz}-derived band with its smaller k_F along the ΓX line compared to the d_{yz}-state in Fig. 1. In the L_2-image we note remnant intensity from the antisymmetric d_{yz}-derived band, which creeps in due to slight relaxation of the symmetry selection rules caused by the tetragonal distortion of STO at low temperatures. Importantly, the ARPES images and the corresponding EDCs in Supplementary Figure 1e and f again reveal the pronounced PDH structure of $A(\omega,\mathbf{k})$ with the LO3-related polaronic hump at ~118 meV.

Supplementary note 2

SX-ARPES data for oxygen-deficient LAO/STO samples

Here, we present SX-ARPES data on oxygen-deficient (OxD) LAO/STO samples to confirm the polaronic origin of the peak-dip-hump spectral structure, and illustrate a possibility to manipulate the EPI through the V_Os. Post-annealing in high pressure of oxygen is an important step in the fabrication of standard LAO/STO samples, which reduces the number of V_Os produced during the non-equilibrium PLD growth[Error! Reference source not found.]. The simplest way to obtain OxD samples is therefore to exclude the last post-annealing step. The main effect of the V_Os in STO is to inject extrinsic charge carriers and thus increase n_s of the interface 2DES (see, for example, Ref. 0).

We have prepared OxD samples using the same growth conditions as described in the Methods section of the main text, but instead of the post-annealing in oxygen the samples were simply cooled down at room temperature at the same oxygen pressure 10^{-4} mbar as used during the deposition. Supplementary Figure 2 shows the SX-ARPES experimental results collected on these samples under the same conditions (experimental geometry, X-ray polarization, resolution and low sample temperature) as the standard oxygen-annealed samples in Fig. 2. The experimental ARPES image in Supplementary Figure 2a demonstrates the expected increase of k_F compared to the standard sample, and fading of the waterfall intensity. Furthermore, the corresponding k_x-integrated spectrum in Supplementary Figure 2b reveals scaling up of broad spectral intensity around -1.2 eV, the spectroscopic signature of the in-gap states induced by the V_Os[3,4], accompanied by dramatic reduction of the polaronic hump. We note that the OxD samples are highly sensitive to X-ray irradiation, acting to multiply V_Os as evidenced by increase of the in-gap spectral intensity and reduction of the polaronic hump with exposure time. The present data have been collected under saturation, achieved after about 15 min of X-ray irradiation with a photon flux of ~10^{13} photons/sec/0.01% bandwidth[5].

Our spectroscopic comparison of the standard and OxD samples, first, identifies the bosonic mode forming the peak-dip-hump spectral structure. To retrieve the hump energy for the OxD sample, we have fitted the experimental k_x-integrated EDC in Supplementary Figure 2b by three Gaussians, representing the in-gap states, hump and the QP peak (whose lineshape is anyway limited by the experimental resolution Gaussian). The fit returns the hump energy 100±20 meV relative to the QP, which is within the error bars identical to the 118 meV for the standard sample. If the hump had the plasmon origin, its energy would scale proportional to $\sqrt{n_s}$. With the n_s values $2.5\cdot10^{14}$ and $7.5\cdot10^{13}$ e/cm^2 determined from the experimental k_F values (see Methods in the main text) for the OxD and standard samples, respectively, the plasmon frequency would change by a factor ~1.7 which is obviously not the case. Therefore, the constant energy of the hump rules out its plasmonic origin. The same conclusion has been made in ARPES experiments on bare STO(100) surfaces[6].

Second, our results suggest a possibility to circumvent the polaronic limit of μ_{2DES} through manipulation of OVs. As explained in the main text, the V_Os inject into the 2DES extrinsic charge carriers[7] which increase the electron screening and thus reduce the EPI strength. We note that the effect of the V_Os is actually beyond the simple doping picture restricted to changing of the band filling within the rigid band shift model. In particular, the V_Os increase the spatial extension of the $d_{xz/yz}$ bands into the STO bulk from ~50 Å for oxygen-annealed samples to ~150 Å and more[8,9,10,11] resulting in predominantly bulk conductivity and loss of the 2D nature of the LAO/STO interface system. Furthermore, the manipulation of the V_Os is complicated by diffusion processes which are hard to precisely control. Further experiments

on OxD-LAO/STO interfaces will allow a better understanding of the role of V_Os and ways to optimize μ_{2DES}.

Supplementary References

1. C. Cancellieri, N. Reyren, S. Gariglio, A.D. Caviglia, A. Fête and J.-M. Triscone. Influence of the growth conditions on the LaAlO3/SrTiO3 interface electronic properties. EPL 91, 17004 (2010).

2. X. Hao, Z. Wang, M. Schmid, U. Diebold and C. Franchini. Coexistence of trapped and free excess electrons in SrTiO3. Phys. Rev, B 91, 085204 (2015).

3. C. Cancellieri, M.L. Reinle-Schmitt, M. Kobayashi, V.N. Strocov, T. Schmitt and P.R. Willmott. Interface Fermi States of LaAlO3/SrTiO3 and Related Heterostructures. Phys. Rev. Lett. 110, 137601 (2013).

4. G. Berner, M. Sing, H. Fujiwara, A. Yasui, Y. Saitoh, A. Yamasaki, Y. Nishitani, A. Sekiyama, N. Pavlenko, T. Kopp, C. Richter, J. Mannhart, S. Suga and R. Claessen. Direct k-Space Mapping of the Electronic Structure in an Oxide-Oxide Interface. Phys. Rev. Lett. 110, 247601 (2013).

5. V.N. Strocov, T. Schmitt, U. Flechsig, T. Schmidt, A. Imhof, Q. Chen, J. Raabe, R. Betemps, D. Zimoch, J. Krempasky, X. Wang, M. Grioni, A. Piazzalunga and L. Patthey. High-resolution soft X-ray beamline ADRESS at the Swiss Light Source for resonant inelastic X-ray scattering and angle-resolved photoelectron spectroscopies. *J. Synchr. Rad.* **17**, 631-643 (2010).

6. Z. Wang, S. McKeown Walker, A. Tamai, Z. Ristic, F.Y. Bruno, A. de la Torre, S. Riccò, N.C. Plumb, M. Shi, P. Hlawenka, J. Sáanchez-Barriga, A. Varykhalov, T.K. Kim, M. Hoesch, P.D.C. King, W. Meevasana, U. Diebold, J. Mesot, M. Radovic and F. Baumberger. Tailoring the nature and strength of electron-phonon interactions in the SrTiO3(001) two-dimensional electron liquid. ArXiV 1506.01191 (2015).

7. X. Hao, Z. Wang, M. Schmid, U. Diebold and C. Franchini. Coexistence of trapped and free excess electrons in $SrTiO_3$. *Phys. Rev, B* **91**, 085204 (2015).

8. P. Delugas, A. Filippetti, V. Fiorentini, D.I. Bilc, D. Fontaine and P. Ghosez. Spontaneous 2-Dimensional Carrier Confinement at the n-Type SrTiO3/LaAlO3 Interface. Phys. Rev. Lett. 106, 166807 (2011).

9. W.-J. Son, E. Cho, B. Lee, J. Lee and S. Han. Density and spatial distribution of charge carriers in the intrinsic n-type LaAlO3-SrTiO3 interface. Phys. Rev. B 79, 245411 (2009).

10. G. Herranz, M. Basletić, M. Bibes, C. Carrétéro, E. Tafra, E. Jacquet, K. Bouzehouane, C. Deranlot, A. Hamzić, J.-M. Broto, A. Barthélémy and A. Fert. High Mobility in $LaAlO_3$/SrTiO3 heterostructures: Origin, dimensionality, and perspectives. *Phys. Rev. Lett.* **98**, 216803 (2007).

11. M. Basletic, J.-L. Maurice, C. Carrétéro, G. Herranz, O. Copie, M. Bibes, É. Jacquet, K. Bouzehouane, S. Fusil and A. Barthélémy, Mapping the spatial distribution of charge carriers in $LaAlO_3/SrTiO_3$ heterostructures. *Nature Mat.* **7**, 621-625 (2008).
